# A novel murine model of late-phase reaction of immediate hypersensitivity

**DOI:** 10.1080/09629359791820

**Published:** 1997-04

**Authors:** S. Facincone, A. L. Pereira De Siqueira, S. Jancar, M. Russo, J. A. Marzagão Barbuto, M. Mariano

**Affiliations:** 1 Department of Immunology Institute of Biomedical Sciences University of São Paulo São Paulo Brazil

## Abstract

We describe here a novel experimental model of late-phase reaction of immediate hypersensitivity developed in mice. It consists of introducing small fragments of heat-coagulated hen egg white into the subcutaneous tissue of mice. After 14 days, animals challenged with purified ovalbumin into the footpad presented an immediate swelling of the paw peaking at 30 min, followed by two peaks of swelling at 6 and 24 h. Histological examination of the paws showed a massive eosinophil infiltration (more than 800 cells/5 microscopic fields). This intense infiltration persisted for more than 14 days after the challenge. Furthermore, in mice immunized with coagulated egg white the delayed swelling of the paws and eosinophilic infiltration were significantly higher than in mice immunized with the classical protocol of ovalbumin in alumen adjuvant. Transfer of lymph node cells obtained from mice implanted with heat-coagulated hen egg white induced footpad swelling and eosinophil infiltration in response to ovalbumin. High levels of ovalbuminspecific IgG1 but not of IgE were detected in the serum of these animals. The advantages of this model for the experimental study of late-phase reaction *per se* and its relevance to the study of allergic diseases such as asthma are discussed.


Mediators of Inflammation, **6**, 127–133 (1997)

### A novel murine model of late-phase reaction of immediate hypersensitivity

S. Facincone, A. L. Pereira de Siqueira, S. Jancar, M. Russo, J. A. Marzagão Barbuto and M. Mariano[CA]

Department of Immunology, Institute of Biomedical Sciences, University of São Paulo, São Paulo, Brazil

[CA]Corresponding Author
Tel: (+55) 11 818 7375
Fax: (+55) 11 818 7224
Email: mmariano@spider.usp.br

**WE describe here a novel experimental model of late-phase reaction of immediate hypersensitivity developed in mice. It consists of introducing small fragments of heat-coagulated hen egg white into the subcutaneous tissue of mice. After 14 days, animals challenged with purified ovalbumin into the footpad presented an immediate swelling of the paw peaking at 30 min, followed by two peaks of swelling at 6 and 24 h. Histological examination of the paws showed a massive eosinophil infiltration (more than 800 cells/5 microscopic fields). This intense infiltration persisted for more than 14 days after the challenge. Furthermore, in mice immunized with coagulated egg white the delayed swelling of the paws and eosinophilic infiltration were significantly higher than in mice immunized with the classical protocol of ovalbumin in alumen adjuvant. Transfer of lymph node cells obtained from mice implanted with heat-coagulated hen egg white induced footpad swelling and eosinophil infiltration in response to ovalbumin. High levels of ovalbumin-specific IgG1 but not of IgE were detected in the serum of these animals. The advantages of this model for the experimental study of late-phase reaction *per se* and its relevance to the study of allergic diseases such as asthma are discussed.**

**Key words:** Asthma, Eosinophil, Hypersensitivity, Inflammation, Late-phase reaction, Mice

### Introduction

Immediate hypersensitivity reactions in the skin, lungs, nose and conjunctiva of atopic individuals, are frequently followed by late-phase reactions (LPR).[1] This type of reaction, whose mechanisms are not yet fully understood, starts within hours of antigen challenge, peaks at 6–12 h and decreases over the next 12–24 h.[2] LPR can be distinguished from delayed-type hypersensitivity (DTH) because it starts earlier compared with DTH and is characteristically infiltrated by eosinophils.[3]

It is generally accepted that LPR underlies the pathogenesis of asthma in man.[4] Nevertheless, the lack of animal models with spontaneous atopy and a clear LPR, limits the investigation of this important phenomenon. To circumvent this limitation, LPR has been investigated in laboratory animals immunized with antigens in different adjuvants.[5–7]

Here we describe a method of inducing a typical and exuberant LPR in mice without the aid of adjuvants. For this, small fragments of coagulated hen egg white were implanted into the subcutaneous tissue of mice. After ovalbumin challenge, the animals exhibited a footpad LPR with persistent eosinophil infiltration that was more intense than that observed in animals immunized with ovalbumin plus alumen (OA-AL).

### Materials and Methods

### Animals

Male or female $F_1$ hybrid mice (BALB/c $\times$ A/J) or BALB/c, A/J and C57BL mice were used in this study.

### Antigens

A beaker containing separated hen egg white, was placed in a water bath at 100°C for 30 min. Fragments of the solidified egg white were washed in distilled water, dehydrated in 100% ethanol for, at least, 24 h and sectioned in small blocks of $4 \times 2 \times 2$ mm (around 40 mg). Before implantation into the animals, the egg white implants (EWI) were re-hydrated by immersion

© 1997 Rapid Science Publishers for 20 min in saline at room temperature. The aggregated ovalbumin (OA) used for the challenge was prepared according to Titus and Chiller.[8] Type II ovalbumin was diluted in saline, pH 7.4 and incubated for 1 h at 80°C. After centrifugation at 3000 × *g* for 10 min, the supernatant was discharged and the pellet ressuspended in sterile saline solution.

### Immunization protocols

Groups of mice were injected s.c. into the base of the tail with 0.2 ml of a suspension of 50 μg/ml of heat-aggregated ovalbumin (OA group) or 0.2 ml of a 1:1 mixture of heat-aggregated OA (100 μg) in $Al[OH]_3$ (4 mg) (OA-AL group). A third group of animals was implanted with heat-coagulated and alcohol treated egg white (EWI group) into the subcutaneous tissue of the dorsal region. After 14 days, immunized animals, and a group of naive mice, were challenged into the hind footpad with 0.05 ml of aggregated OA (20 mg/ml). Footpad swelling was measured with the aid of a gauge caliper (Mitutoyo). The swelling induced by OA in non-immunized animals was subtracted from that induced by OA in immunized animals at all experimental times.

### Titration of anaphylactic antibodies by ELISA

IgG1 and IgE antibodies against OA were titrated by ELISA in the plasma of mice that received EWI, OA-AL and in the plasma of non-immunized animals. For IgG1 determination, microplates were coated overnight at 4°C with 10 μg/ml of OA (Grade V) in carbonate buffer at pH 9.6. For IgE titration, the plates were coated with LO-ME-2 monoclonal antibody (rat IgG2a–k specific for mouse IgE). After washing five times with PBS supplemented with 0.1% Tween 20, they were blocked with 1% gelatin in PBS for 2 h at 37°C. The plates were washed and serial dilution of the plasma were added. After incubation for 1 h at 37°C the plates were washed and peroxidase labelled LO-MG-1 monoclonal antibody (rat IgG2a–k specific for mouse IgG1) for IgG1 determination or peroxidase labelled OA for IgE (1 μg/ml) were added and incubated for 1 h. After several washings, orthophenyldiamine (OPD), 0.4 mg/ml, diluted in citrate/phosphate buffer, pH 5.6 and $H_2O_2$ were added. The reaction was stopped with 50 μl of $H_2SO_4$ 1 M and the optical density determined at 492 nm. The monoclonal antibodies to mouse IgG1 (LO-MG-1PO) and IgE (LO-ME-2) were kindly supplied by Dr H. Bazin (UCL, Belgium).

### Titration of IgE antibodies by PCA

The antigen specific IgE antibody content of plasma was estimated by PCA method according to Mota and Wong.[9] Intradermal injection of serial dilution of the mice immune sera were made in the shaved skin of Wistar rats. Forty-eight hours afterwards the animals were injected i.v. with 1 ml of 0.25% solution of Evans blue dye in saline solution containing 0.5 mg of ovalbumin. Thirty minutes later, the animals were killed with an overdose of ether, the skin inverted and the lesion's diameter measured. PCA titres represent the highest dilution of serum yielding a lesion of more than 5 mm in diameter.

### Eosinophil peroxidase activity assay

The EPO activity in bone marrow cells was determined with a colorimetric assay as described by Strath *et al.*[10] Briefly, bone marrow cells were obtained from femurs by flushing them with 1 ml of PBS. The red cells were lysed and the cell suspension adjusted to the desired concentration ($10^4$ and $10^5$ cells per well). One hundred microlitres of the cell suspension were added to 96-well plates. The plates were centrifuged at 200 × *g* at 4°C for 10 min and the supernatants were discharged. A substrate solution (100 μl) containing 0.1 mM orthophenylene diamine dihydrochloride (OPD) in 50 mM Tris-HCl with 0.1% Triton X-100 and 1 mM hydrogen peroxide was added to each well. The reaction was stopped after 30 min with 50 μl of 4 M sulphuric acid and the absorbance of the samples determined at 492 nm. Each value represents the mean ± SD from five individual mice.

### Transference of lymph node cells

Axillary and inguinal lymph node cells were harvested from animals implanted with EWI or from non-immunized mice. The cells from each group of animals were pooled, counted and adjusted to the concentration of $9 \times 10^7$ cells/ml in RPMI-1640 (Sigma). These cells were either diluted in RPMI alone or in RPMI containing crystallized OA and adjusted to a final concentration of $9 \times 10^6$ cells and 0.24 mg of OA/ml. A volume of 0.05 ml of these suspensions was injected into the hind footpad of different groups of naive mice. Two other groups of mice received the same number of cells from immunized or non-immunized mice without the addition of OA. Finally, one group was injected with saline and another with OA

in RPMI at the same concentration as above. Footpads were measured after different time intervals, removed after 48 h and processed for histological analysis.

### Histology and electron microscopy

Fragments of footpad lesions were fixed in neutral 10% formalin and processed for routine histology. Histological sections were stained by the methods of HE or Litt for eosinophil identification. The relative number of cells in the lesions was evaluated using an integrator eye piece (Zeiss) with 25 hits. The mean of 10 microscopic fields (obj. 100×) was obtained for each histological preparation.

Fragments of footpad lesions were fixed in 2% buffered glutaraldehyde and 2% paraformaldehyde in 0.1 M sodium cacodylate buffer (pH 7.2). After fixation with 2% $OsO_4$, fragments were dehydrated and embedded in Spurr resin. Thin sections were stained with uranyl acetate and lead citrate and examined with a Jeoll 100 CXII transmission electron microscope.

### Statistical analysis

Data were analysed statistically using a microcomputer program and an analysis of variance, followed by Tukey's multiple comparison tests. The statistics have been performed on the absolute values.

### Results

### Footpad swelling after challenge

Mice were challenged with OA in the footpad, 14 days after immunization, and the footpad swelling was measured (Fig. 1). Animals immunized with EWI or with OA-AL presented an immediate swelling and a late phase response (LPR) which showed maximal intensity at 24 h, while mice immunized with aggregated OA, presented a mild swelling of the paws 30 min after OA challenge and no swelling thereafter. The EWI groups showed a significantly more intense LPR than the OA-AL group.

Similar results were obtained when BALB/c, A/J and C57BL mice were used (data not shown).

### Histopathology of footpad lesions

Histological examination of the lesions induced after antigen challenge varied according to the protocols used. The lesions observed in animals from the OA-AL group is composed by mono-

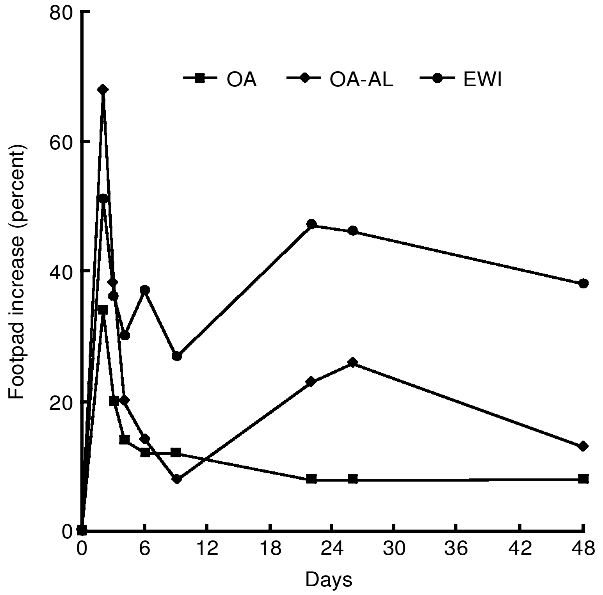

FIG. 1. Time-response curves of footpad swelling obtained in mice immunized with OA, OA-AL or with EWI and challenged in the footpad with aggregated OA 14 days later (n = 6). Results are expressed as percentage increase in paw volume over that induced by OA injection in non-immunized mice. Data represent the mean ± SEM. Differences observed between OA-AL and EWI immunization are statistically significant ( $p < 0.005$) at all times examined.

nuclear cell and eosinophils arranged in an homogeneous distribution. Conversely, in animals from the EWI group, a central area composed by mononuclear cells surrounded by intense eosinophil infiltration was observed. Figure 2 shows the differences in cell type infiltration in 24 h lesions induced in OA, OA-AL or EWI groups. The total number of inflammatory cells as well as the number of eosinophils was significantly higher in lesions of the EWI group compared with the OA-AL or OA groups. The number of neutrophils in the lesions was low and did not vary among the immunization protocols.

This eosinophil infiltration persisted for 21 days in the site of antigen inoculation in the EWI groups (Fig. 3).

The relative number of mononuclear cells in the lesions remained constant during the first week, but increased significantly by the third week. This relative increase reflects the change in total cell numbers observed at this time (Fig. 3).

Typical histological characteristics of lesions induced by OA inoculation in EWI mice are shown in Fig. 4A and B.

The ultrastructural analysis confirmed the intense eosinophilic infiltration of the lesions in EWI animals. Yet, macrophages with morphologic characteristics of activated cells and a few

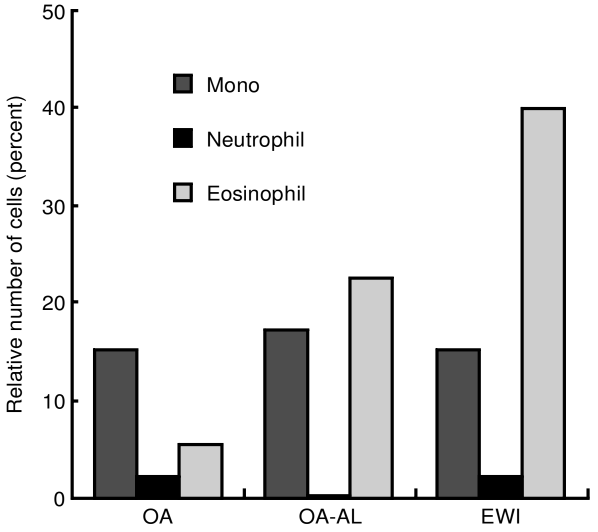

FIG. 2. Relative number of cells infiltrating footpad lesions induced by the injection of aggregated OA in mice immunized by different protocols ($n = 6$). Data were obtained with the aid of an integrator eye piece. The higher number of eosinophils in lesions induced in animals immunized with EWI is statistically significant when compared with animals immunized with OA alone or OA-AL. No difference was detected regarding the number of monocytes and neutrophils ($p < 0.005$).

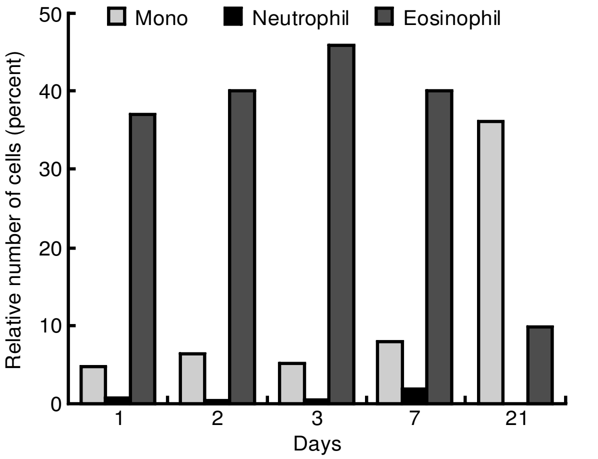

FIG. 3. Time course of cell infiltration into the footpad lesions induced by the injection of aggregated OA in animals previously immunized with EWI ($n = 8$). Data were obtained with the aid of an integrator eye piece. Up to 7 days, the number of eosinophils was significatively higher compared with the number of neutrophils and mononuclear cells. This difference disappeared in lesions examined after 21 days of challenge ($p < 0.005$).

lymphoid cells were also present. Basophils were not found in these lesions (Fig. 5).

### Antibody isotypes

The plasma of mice immunized with EWI or OA-AL were screened for anti-OA IgG1 or IgE isotypes by ELISA. OA specific IgG1 antibodies were detected in both groups and were significantly higher (2.6 times) in the EWI group (Fig. 6). The IgE isotype was measured using a reverse (IgE capture) ELISA to avoid interference of non-IgE mast cell sensitizing antibodies. OA specific IgE antibodies were not detected either in the OA-AL or in the EWI group (data not shown). However, by PCA reaction low levels of OA-specific IgE were detected in the plasma, the titre being 1:5 in both groups (median of six sera from each group).

### Transference of lymph node cells

Lymph node cells from animals that received EWI, were injected into the footpad of naive mice as described in Materials and Methods. As shown in Fig. 7, the lymph node cells from EWI immunized animals stimulated with OA induced a significantly higher paw swelling than that of animals which received these cells without OA stimulation. Cells from non-immunized animals were unable to induce an enhanced response to OA (data not shown). The histology of footpad lesions 24 h after cell transfer, showed an inflammatory response composed mainly by mononuclear cells in animals which received lymph node cells from EWI immunized animals without OA stimulation. The addition of OA to this cell suspension, evoked a marked infiltration of eosinophils. The other controls either did not show any inflammatory response or a mild mononuclear infiltrate (data not shown).

The injection of OA into the footpad of mice which received serum from EWI immunized mice did not induce significant increase in the footpad of these animals (data not shown).

### EPO activity in bone marrow cells

Since analysis of eosinophil activation in the footpad lesions is limited by technical reasons we determined the specific peroxidase activity (EPO) in the bone marrow. It can be seen in Fig. 8 that EPO activity was higher in bone marrow cells harvested from the EWI compared with the OA-AL group.

### Discussion

Our results show that the implantation of fragments of coagulated egg white into the subcutaneous tissue of mice followed by OA challenge, results in a LPR similar to that described in atopic patients.[1] LPR in EWI animals is notably more intense than that obtained in animals immunized with OA-AL, with an additional peak at 6 h after challenge (not

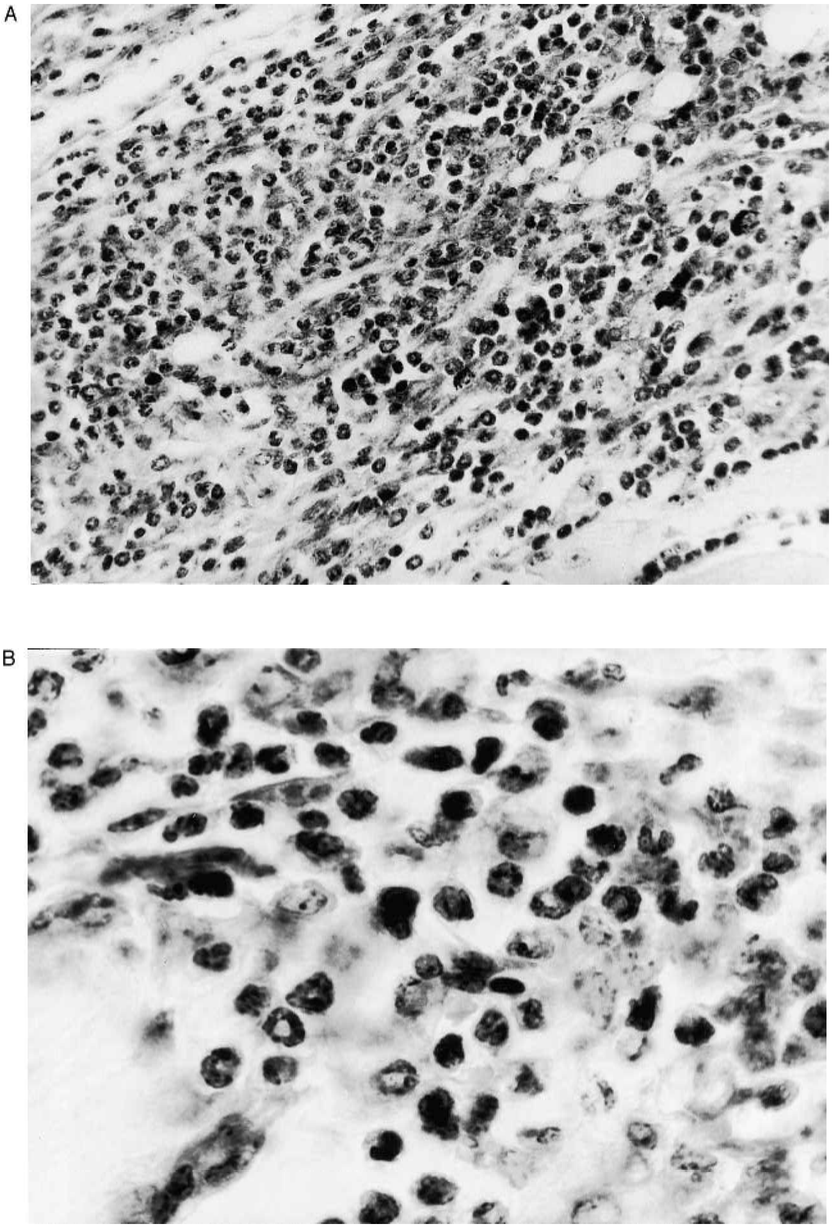

FIG. 4. Photomicrography of a lesion induced by aggregated OA injection into the footpad of mice immunized with EWI 24 h after challenge. (A) A dense cellular infiltrate predominantly composed by eosinophils can be observed. (B) shows the cellular infiltrate in a closer detail. H.E., 250 × (A) and 400 × (B).

observed in OA-AL animals). Moreover, the inflammatory infiltrate obtained in EWI animals was characterized by a clear predominance of eosinophils over other cell types, which lasted for more than 2 weeks. Again, this feature resembles human disorders, where 24–48 h after antigen challenge, a large number of eosinophils and mononuclear cells are recruited to the site of antigen inoculation in sensitized individuals.[3]

The degree of activation of eosinophils from within the lesions could not be investigated in this model. However, the amount of eosinophil peroxidase activity detected in bone marrow cells was higher in the EWI group compared with animals sensitized with OA-AL. It is known that IL-5 is a selective cytokine that promotes eosinophil production and release from the bone marrow, migration to the tissues, activation and survival.[11] Thus, the strong eosinophilic infiltration and activation observed in the EWI model may be ascribed to enhanced IL-5 production.

Also observed was a pronounced infiltration of mononuclear cells but not of neutrophils in these lesions. Moreover, macrophages, as characterized by ultrastructural analysis, showed a typical morphology of cells in different stages of

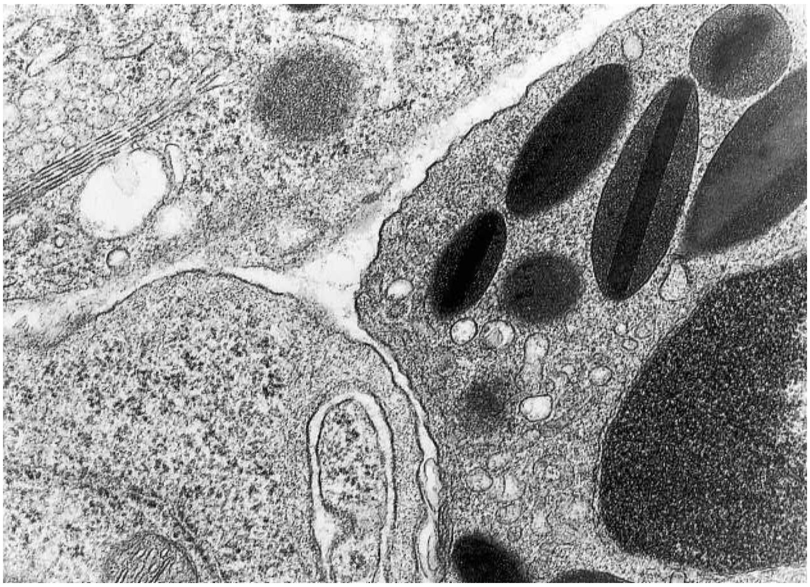

FIG. 5. Electron micrography of a lesion induced as in Fig. 4 showing an eosinophil, a macrophage and a lymphoid cell in close contact. 10 000×.

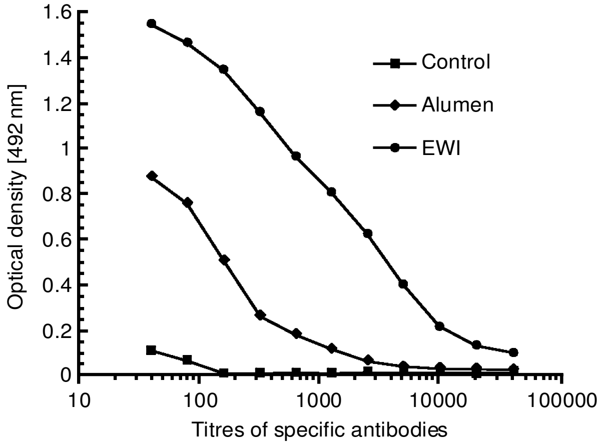

FIG. 6. OA specific IgG1 antibody produced by EWI or OA-AL immunization. Isotype concentrations in immune and non-immune plasma (control) obtained 13 days after immunization and titrated by ELISA. Results represent the mean of five sera from each group.

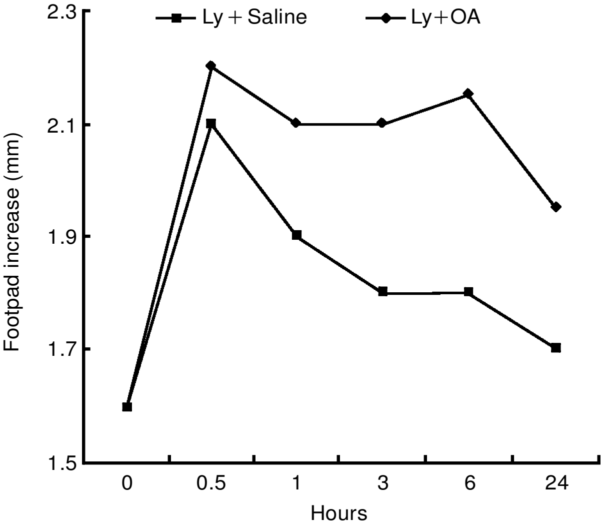

FIG. 7. Lymph node cells from animals immunized with EWI were collected (n = 10), stimulated or not with OA and injected into the footpad of naive animals (n = 6) (see controls in Materials and Methods). The increase in footpad diameter was recorded. The transference of EWI lymph node cells stimulated with OA (Ly + OA) induced a persistent increase in volume which was significantly higher (starting after 6 h) when compared with animals which received EWI lymph node cells without OA stimulation (Ly + saline) ($p < 0.005$).

activation. A few lymphocytes in close contact with macrophages and eosinophils could be observed but basophils were absent. These observations and the fact that adoptive transfer of lymphocytes from EWI mice to naive animals reproduce the lesions support the hypothesis that T cells have a role in this phenomenon. Iwamoto *et al.*[12] showed that eosinophil infiltration into the subcutaneous tissue of OA-sensitized mice was biphasic and that the second peak (24–48 h) of antigen-induced eosinophil recruitment was dependent on $CD4^+$ T cells and IL-5 production.[12,13] In our model, the participation of T cell subsets awaits further characterization.

It is also noteworthy that both types of immunization procedures, with EWI or OA-AL, induced specific IgG1 production although the EWI protocol induced much higher levels of this isotype. Mouse IgG1 isotype is the non-IgE mast cell sensitizing antibody of this animal

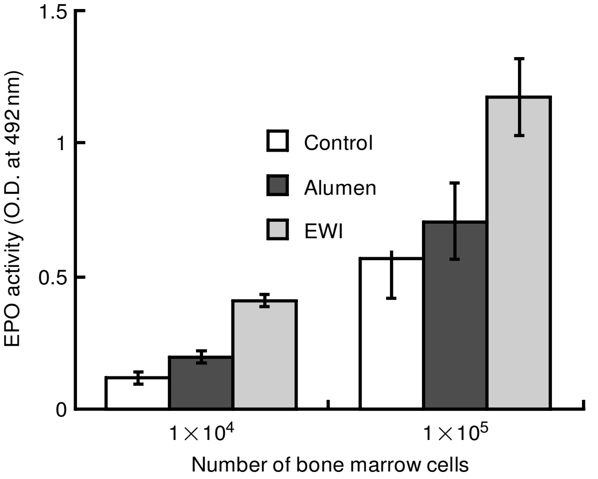

FIG. 8. EPO activity of $10^4$ or $10^5$ bone marrow cells (n = 6) from EWI or OA-Alumen immunized mice, 3 days after intratracheal antigen challenge. Results are expressed as O.D. at 492 nm and represent the mean ± SD.

species.[14] Both protocols of immunization induced similar levels of IgE production. The production of this isotype was very low and was only detected by PCA reaction. The lack of booster injection of antigen would explain the low level of IgE in the AO-AL group.

The reason why EWI induces this peculiar pattern of hypersensitivity response remains to be elucidated. Antigen immobilization might be one of the facilitating factors for the induction of LPR. Wieslander *et al.*[15] induced secondary allergic response to OA in guinea pigs by coupling the antigen covalently to Sepharose beads. Similarly, adjuvants such as alumen or Freund's adjuvant also retain antigen within the injection point. However, adjuvants add unknown variables to the immunization process and our protocol, being able to induce LPR without these factors, could facilitate the unravelling of the immunological mechanisms of this reaction. The possibility that the EWI might have some chemical components with adjuvant properties, was considered. To test this hypothesis, grade II purified OA solidified by heating was implanted into the subcutaneous tissue of the animals. This type of immunization was as effective as EWI to induce a LPR in the footpad of the animals after challenge with aggregated OA (data not shown). These results demonstrate that immobilization of the antigen rather than chemical components of the egg white is the determining factor which modulate the immune response to ensure the observed LPR after challenge.

In conclusion, we describe here that the implantation of coagulated egg white can induce a typical LPR in the mouse, an animal largely manipulated for immunological investigation. Therefore, this new model for the study of the LPR carries the potential to considerably further the understanding of this phenomenon.

### References

1. Frew AJ, Key AB. Eosinophil and T-lymphocytes in late-phase allergic reaction. *J All Clin Immunol* 1990; **85**: 533–539.
2. Zweiman B. The late-phase reaction: role of IgE, its receptor and cytokines. *Curr Opinions Immunol* 1993; **5**: 950–955.
3. Gaga M, Frew AJ, Varney AV, Key AB. Eosinophil activation and T-lymphocyte infiltration in allergen-induced late phase skin reaction and classical delayed-type hypersensitivity. *J Immunol* 1991; **147**: 816–822.
4. Seminario MC, Gleich GJ. The role of eosinophils in the pathogenesis of asthma. *Curr Opinions Immunol* 1994; **6**: 860–864.
5. Fujimaki H, Ozawa M, Imai T, Kubota K, Watanabe N. Adjuvant effect of aluminum silicate on IgE and IgG1 antibody production in mice. *Int Arch Allergy Appl Immunol* 1994; **75**: 351–356.
6. Pretolani M, Ruffié C, Joseph D, *et al.* Role of eosinophil activation in the bronchial reactivity of allergic guinea pigs. *Am J Crit Care Med* 1994; **149**: 1167–1174.
7. Mauser PJ, Pitman AM, Fernandez X, *et al.* Effect of an antibody to interleukin-5 in a monkey model of asthma. *Am J Resid Crit Care Med* 1995; **152**: 467–472.
8. Titus RG, Chiller JM. A simple and effective method to assess murine delayed-type hypersensitivity to proteins. *J Immunol Methods* 1981; **45**: 65.
9. Mota I, Wong D. Homologous and heterologous passive cutaneous anaphylactic reaction. *Life Sci* 1969; **8**: 813–820.
10. Strath M, Warren DJ, Sanderson L. Detection of eosinophils using an eosinophil peroxidase assay. Its use as an assay for eosinophil differentiation factors. *J Immunol Methods* 1985; **83**: 209–304.
11. Yamaguchi Y, Suda T, Suda J, Eguchi M, Miura Y, Harad N, Taminaga A, Takatsu K. Purified interleukin 5 supports the terminated differentiation and proliferation of eosinophilic precursors. *J Exp Med* 1988; **167**: 43–56.
12. Iwamoto I, Tomoe S, Tomioka H, Takatsu K, Yoshida S. Role of CD4+ T lymphocytes and interleukin-5 in antigen-induced eosinophil recruitment into the site of cutaneous late-phase reaction in mice. *J Leuk Biol* 1992; **52**: 572–578.
13. Kaneko M, Hitoshi Y, Takatsu K, Matsumoto S. Role of interleukin-5 in local accumulation of eosinophils in mouse allergic peritonitis. *Int Arch Allergy Appl Immunol* 1991; **96**: 41–45.
14. Nussensweig RS, Merryman C, Benacerraf C. Electrophoretic separation and properties of mouse antihapten antibodies involved in passive cutaneous anaphylaxis and passive hemolysis. *J Exp Med* 1964; **120**: 315–328.
15. Wieslander P, Andersson M, Linden M, *et al.* Importance of particulate antigen for the induction of dual bronchial reaction in guinea-pigs. *Agents and Action* 1985; **16**: 37–38.

ACKNOWLEDGEMENTS. The authors are pleased to acknowledge Dr Paulo Abrahamson for electron microscopy analysis of the lesions and to Dr Maria Fernanda Macedo Soares for the isotypes titration. We are also thankful to Dr Mahasti S. de Macedo for helpful discussions. Financial support by the state granting agency (FAPESP) and by the National Research Council (CNPq-PADCT). S.F. is a post-graduate student from the Department of Pathology, School of Veterinary Medicine, USP.

**Received 26 November 1996;**
**accepted in revised form 9 January 1997**
